# Changes in rural older adults’ sedentary and physically-active behaviors between a non-snowfall and a snowfall season: compositional analysis from the NEIGE study

**DOI:** 10.1186/s12889-020-09343-8

**Published:** 2020-08-17

**Authors:** Shiho Amagasa, Shigeru Inoue, Hiroshi Murayama, Takeo Fujiwara, Hiroyuki Kikuchi, Noritoshi Fukushima, Masaki Machida, Sebastien Chastin, Neville Owen, Yugo Shobugawa

**Affiliations:** 1 Department of Preventive Medicine and Public Health, Tokyo Medical University, 6-1-1 Shinjuku, Shinjuku-ku, Tokyo, 160-8402 Japan; 2 Institute of Gerontology, The University of Tokyo, 7-3-1 Hongo, Bunkyo-ku, Tokyo, 113–8656 Japan; 3 Department of Global Health Promotion, Tokyo Medical and Dental University, 1-5-45, Yushima, Bunkyo-ku, Tokyo, Japan; 4 School of Health and life Science, Institute of Applied Health Research, Glasgow Caledonian University, 70 Cowcaddens Road, Glasgow, UK; 5 Department of Sport and Movement Science, Ghent University, 9000 Ghent, Belgium; 6 Behavioral Epidemiology Laboratory, Baker Heart & Diabetes Institute, Level 4, 99 Commercial Road, Melbourne, Vic 3004 Australia; 7 Centre for Urban Transitions, Swinburne University of Technology, PO Box 218, Hawthorn, Melbourne, Australia; 8 Division of International Health, Niigata University Graduate School of Medical and Dental Sciences, 1-757, Asahimachi-dori, Niigata City, 951-8510 Japan; 9 Department of Active Ageing, Niigata University Graduate School of Medical and Dental Sciences, 1-757, Asahimachi-dori, Niigata City, 951-8510 Japan

**Keywords:** Accelerometry, Aging, Environment, Exercise, Sedentary lifestyle

## Abstract

**Background:**

Levels of physical activity change throughout the year. However, little is known to what extent activity levels can vary, based on accelerometer determined sedentary and physically-active time. The aim of this longitudinal study was to examine older adults’ activity changes from a non-snowfall season to a subsequent snowfall season, with consideration of the co-dependence of domains of time use.

**Methods:**

Participants were 355 older Japanese adults (53.1% women, aged 65–84 years) living in a rural area of heavy snowfall who had valid accelerometer (Active style Pro HJA-750C) data during non-snowfall and snowfall seasons. Activity was classified as sedentary behavior (SB), light-intensity PA (LPA), and moderate-to-vigorous PA (MVPA). Compositional changes from the non-snowfall to the snowfall season were analyzed using Aitchison’s perturbation method. The ratios of each component in the composition, such as [SBsnow/SBnon-snow, LPAsnow/LPAnon-snow, MVPAsnow/MVPAnon-snow] for seasonal changes, were calculated and were then divided by the sum of these ratios.

**Results:**

In men, the percentages of time spent in each activity during the non-snowfall/snowfall seasons were 53.9/64.6 for SB; 40.8/31.6 for LPA; and 5.3/3.8 for MVPA; these corresponded to mean seasonal compositional changes (∆SB, ∆LPA, ∆MVPA) of 0.445, 0.287, and 0.268 respectively. In women, the percentages of time spent in each activity during the non-snowfall/snowfall seasons were 47.9/55.5 for SB; 47.9/41.0 for LPA; and 4.2/3.5 for MVPA; these corresponded to mean seasonal compositional changes (∆SB, ∆LPA, ∆MVPA) of 0.409, 0.302, and 0.289 respectively. The degree of seasonal change was greatest in men.

**Conclusions:**

In older adults, activity behaviors were changed unfavorably during snowfall season, particularly so for men. The degree of seasonal change was greatest for SB. Development of strategies to keep rural older adults active during the snowfall season may be needed for maintaining a consistently-active lifestyle for their health.

## Background

Global physical activity guidelines state that older adults should do at least 150 min per week of moderate-intensity physical activity, at least 75 min vigorous-intensity physical activity, or an equivalent combination of both, in bouts lasting 10 min or more [1]. Physical inactivity increases the risk of many adverse health outcomes including mortality, cardiovascular disease, type 2 diabetes, several types of cancer, and cognitive decline [2–6]. In recent years, there has been significant growth in research investigating the detrimental health effects of sedentary behavior (SB; put simply, too much sitting) [7, 8]. Reducing SB and increasing physical activity are key ingredients of initiatives to reduce the global burden of non-communicable diseases [2, 9].

There is a growing body of evidence identifying the importance of natural environments (the attributes of which can vary greatly by season) as well as built environments as influences on physical activity [10, 11]. One systematic review of 37 studies from eight different countries showed that levels of physical activity tended to be lowest during winter [10]. To date, however, most of these studies have relied on self-report physical activity assessments and have not focused on older adults [10]. Objective assessment (via accelerometers) can now overcome problems of recall and reporting bias and allow more accurate and finer-grained assessments of activity behavior patterns: SB, light-intensity physical activity (LPA), and moderate-to-vigorous physical activity (MVPA) [12]. There is limited evidence on how season affects device-based activity behaviors [13–15]. Compositional data analysis (CoDa) allows consideration of the co-dependence of time spent in all behaviors arising within a day or other fixed period [16, 17]. To date, no previous study has investigated seasonal changes of activity behaviors, taking time spent in each activity behavior into account.

We compared times spent in accelerometer-measured activity behaviors during a non-snowfall and a snowfall season in a sample of community-dwelling older Japanese adults, using the CoDa approach. We also explored which activity behaviors were most affected by season.

## Methods

### Study sample and data collection

We used longitudinal data from the Neuron to Environmental Impact across Generations study (NEIGE study). The methods of this study are described in previous study [18]. Participants were older adults without long-term care living in Tokamachi city, Japan. Tokamachi is a rural city, officially registered as a heavy snowfall region during winter, located in the southernmost region of Niigata Prefecture. Participants were from two areas: Matsunoyama (mountain) area or Tokamachi area (downtown). The mountain area (2.89 m maximum snow depth) had more snow than the downtown area (1.97 maximum snow depth) during winter [18]. The average temperature at Tokamachi city in February 2018 was − 0.9 °C (lowest − 8.3 °C, highest 9.1 °C) [19].

Briefly, in 2017, a total of 1346 residents were recruited from a resident registry, using stratified random sampling. In the non-snowfall season (autumn) of 2017, we conducted a questionnaire survey and health examination of 527 older adults who agreed to enrollment in the NEIGE study; at the same time, they were asked to wear an accelerometer. Of these participants, 381 agreed to also wear an accelerometer during the snowfall season (winter) of 2018.

### Accelerometer-measured activity behaviors

Habitual time spent in activity behaviors were evaluated by Active style Pro HJA-750C (Omron Healthcare, Kyoto, Japan). Active style Pro is a validated accelerometer [20–23] and comparable to the devices most commonly used in studies conducted in Western countries [24, 25]. Its measurement algorithm has been explained in detail elsewhere [20, 21]. Participants were instructed to wear an accelerometer over the waist on an elasticated belt for seven consecutive days (except during sleep and water-based activities) during snowfall and non-snowfall season, respectively. In the survey during snowfall season, participants were mailed an accelerometer. No acceleration signal detected for longer than 60 consecutive minutes was defined as “non-wear”. Participants with a wear time corresponding to at least 10 h during waking time per day [26], collected over four or more valid days were included in the analysis [27]. The data were collected in 60-s epochs. Activity behaviors were classified into three intensity categories based on metabolic equivalents (METs): SB (≤1.5 METs), LPA (1.6–2.9 METs), and MVPA (≥3.0 METs) [28, 29].

### Sociodemographic, biological, and psychological factors

Participants reported their age, gender, living arrangement (with others/ alone), and self-rated health (very good/ good/ fair/ poor) in autumn. We classified participants between the ages of 65 and 74 years as young-old, and those between ages 75 and 84 years as old-old. Body mass index (BMI) was calculated from height and weight (kg/m^2^) measured by body composition analyzer MC-780A (TANITA corporation, Tokyo, Japan).

### Statistical analyses

All analyses were performed using R version 3.5.2 (R Foundation for Statistical Computing, Vienna, Austria). We used R package “compositions” for CoDa approach. For all analyses, *p*-values <.05 were considered statistically significant. Analyses were applied stratified by gender since activity behavior patterns were significantly different between men and women.

The Chi-Square test or t test was performed to compare participant characteristics. McNemar’s test was used to compare non-snowfall and snowfall season’s proportions of those adhering to physical activity guidelines (i.e. ≥150 min/week of MVPA in bouts of at least 10 min) [1]. A 10 min-bout MVPA was defined as 10 or more consecutive minutes above the moderate intensity threshold, with the allowance of 1–2 min interruption intervals [27].

We described activity behaviors during the non-snowfall and snowfall season using CoDa approach. As all participants spent some time in every behavior there was no need for a method to deal with zeros. Compositional changes [∆SB, ∆LPA, ∆MVPA] were then determined by Aitchison’s perturbation method [30–32]. The ratios of each component in the composition, such as [SBsnow/SBnon-snow, LPAsnow/LPAnon-snow, MVPAsnow/MVPAnon-snow] for seasonal changes, were calculated and were then divided by the sum of these ratios. An equal composition of these three activities in the non-snowfall and snowfall seasons would result in a compositional change of [1/3, 1/3, 1/3]. Compositional changes were plotted as ternary diagrams, with some significant guide values illustrated.

## Results

### Participant enrollment and characteristics

Of the 381 older adults who agreed to wear an accelerometer during both the non-snowfall and snowfall seasons (response rate: 28.3%), 26 were excluded for; not meeting wearing time criteria (*n* = 23), bone fracture (*n* = 1), unreturned accelerometer (n = 1) and malfunction (n = 1). The analytic sample was 355 who had valid accelerometer data.

When compared to participant characteristics between those who had valid accelerometer data in snowfall season and those who did not, a significant difference was found in age groups (young-old: 73.7%, old-old: 59.8%, Chi-Square test). Participants who participated in the survey in the snowfall season were significantly more physically active (approx. 10 more minutes of MVPA per day) than those who did not (t-test).

Table 1 presents the characteristics of the participants. The mean (SD) age was 72.9 (5.4) years (52.4% women) and most of the participants were living with others, < 25.0 kg/m^2^ BMI, and good perceived health. Compared to women, men were more likely to be from the mountain area, be living with others and to have attained ≥150 min/week of MVPA. There were no significant seasonal differences in proportion of those adhering to global physical activity guidelines (non-snowfall season: overall 24.2%, men 33.1%, women 16.1%, snowfall season: overall 21.7%, men 27.2%, women: 16.7%).

**Table 1:** Participant’s characteristics at baseline (non-snowfall season)

	Overall (*n* = 355)		Men (*n* = 169)		Women (*n* = 186)		
	Mean ± SD / n (%)		Mean ± SD / n (%)		Mean ± SD / n (%)		P-value
Age, yrs.	72.9 ± 5.4		72.8 ± 5.5		73.1 ± 5.3		0.703
Age categories							0.912
Young-old (65–74 yrs.)	225	(63.4)	108	(63.9)	117	(62.9)	
Old-old (≥75 yrs.)	130	(36.6)	61	(36.1)	69	(37.1)	
Area of residence							**0.014**
Mountain area	160	(45.1)	88	(52.1)	72	(38.7)	
Downtown	195	(54.9)	81	(47.9)	114	(61.3)	
Living arrangement							**0.002**
Living with others	320	(90.1)	161	(95.3)	159	(85.5)	
Living alone	35	(9.9)	8	(4.7)	27	(14.5)	
BMI, kg/m^2^	22.6 ± 2.8		23.1 ± 2.4		22.2 ± 3.1		**0.002**
BMI categories							0.782
Normal (< 25.0 kg/m^2^)	291	(82.0)	140	(82.8)	151	(81.2)	
Obese (≥25.0 kg/m^2^)	64	(18.0)	29	(17.2)	35	(18.8)	
Perceived health							0.392
Good (very good/ good)	305	(85.9)	148	(87.6)	157	(84.4)	
Poor (fair/ poor)	50	(14.1)	21	(12.4)	29	(15.6)	

*P*-value was calculated using t test or chi-square test, as appropriate

### Activity behaviors in snowfall and non-snowfall season

**Fig. 1:**
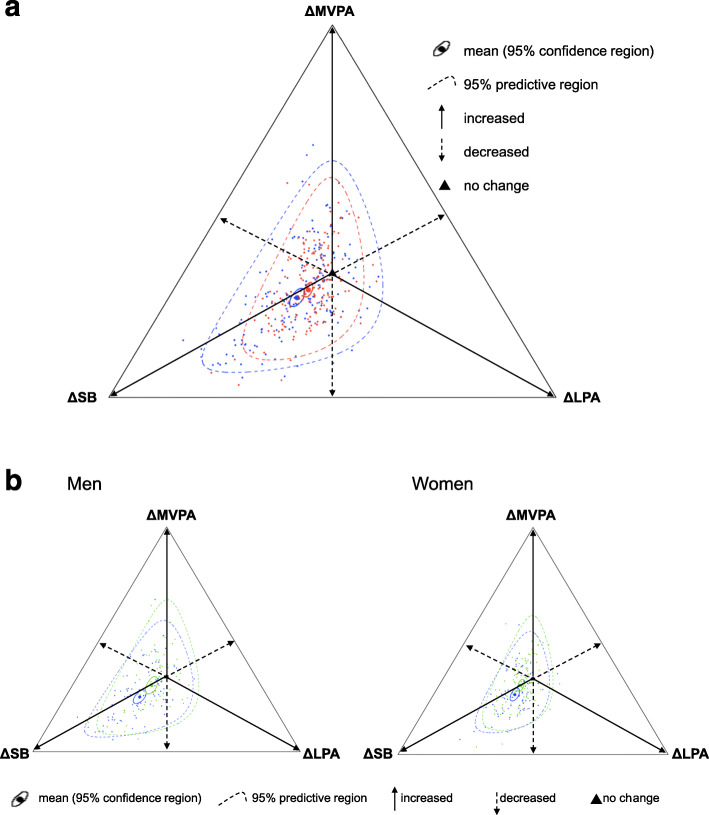
Changes in sedentary behavior (SB), light intensity physical activity (LPA) and in moderate-to-vigorous physical activity (MVPA) from the non-snowfall to snowfall season in rural-dwelling older Japanese adults. **a** Stratified by gender (blue: men, red: women), **b** Stratified by gender and residential area (blue: mountain area, green: downtown). Abbreviation: SB; sedentary behavior, LPA; light-intensity physical activity, MVPA; moderate-to-vigorous physical activity

Mean accelerometer wear time was 891.7 min/day in non-snowfall season and 886.8 min/day in snowfall season. Older adults spent most of their time on SB and LPA. Table 2 presents the descriptive statistics of time spent in each activity behavior. In men, time spent in each activity (SB, LPA, and MVPA) was 53.9, 40.8, and 5.3%, respectively during the non-snowfall season, and 64.6, 31.6, and 3.8%, respectively during the snowfall season, corresponding to mean seasonal change of 0.445 for SB, 0.287 for LPA, and 0.268 for MVPA (Fig. 1). Since larger distance from 1/3 indicates greater change in time spent in activity, the largest seasonal change was observed in SB, compared to LPA and MVPA. In women, time spent in each activity was 47.9, 47.9, and 4.2%, respectively during non-snowfall season while that was 55.5, 41.0, and 3.5%, respectively during snowfall season, corresponding to mean seasonal change of 0.409 for SB, 0.302 for LPA, and 0.289 for MVPA. Significant unfavorable seasonal changes in activity behaviors were observed in both men and women, but the degree of change was larger in men in every behavior if we contrast the changes (the ratios) between men and women. Similar findings were observed after stratification by residential area, but the degree of change in activity behaviors was larger in mountain area than in downtown, regardless of gender.

**Table 2:** Compositional (geometric) means of time spent in activity behaviors during the non-snowfall and snowfall season

	Non-snowfall season, min/day (% wear time)				Snowfall season, min/day (% wear time)			
	Wear time	SB	LPA	MVPA	Wear time	SB	LPA	MVPA
Overall	891.7	430.5 (50.8)	377.1 (44.5)	39.8 (4.7)	886.8	507.9 (60.0)	308.3 (36.4)	30.7 (3.6)
Men	869.2	443.2 (53.9)	335.2 (40.8)	43.2 (5.3)	877.4	541.5 (64.6)	264.6 (31.6)	31.7 (3.8)
Mountain area	875.1	412.6 (50.2)	362.9 (44.1)	47.1 (5.7)	867.9	528.4 (63.8)	269.6 (32.5)	30.7 (3.7)
Downtown	864.1	479.0 (58.0)	307.5 (37.2)	39.2 (4.8)	890.8	556.0 (65.6)	259.2 (30.6)	32.9 (3.9)
Women	912.6	419.2 (47.9)	419.6 (47.9)	36.9 (4.2)	895.3	479.2 (55.5)	354.2 (41.0)	29.8 (3.5)
Mountain area	935.7	404.0 (45.1)	451.2 (50.4)	40.5 (4.5)	903.7	474.2 (54.6)	365.5 (42.1)	28.7 (3.3)
Downtown	900.0	429.1 (49.6)	400.8 (46.3)	34.9 (4.0)	892.4	482.4 (56.1)	347.2 (40.4)	30.5 (3.5)

*Abbreviation*: *SB* sedentary behavior, *LPA* light-intensity physical activity, *MVPA* moderate-to-vigorous physical activity

## Discussion

We identified seasonal differences of accelerometer determined activity behaviors in community-dwelling older adults in a rural snowfall area, using the CoDa approach. Older adults had more unfavorable activity behavior patterns during the snowfall season compared to non-snowfall season, with the magnitude of these differences greater in men. No significant seasonal differences were found in the proportions adhering to global physical activity guidelines.

Our findings are consistent with previous studies using self-report [33, 34] and pedometer/ accelerometer [13–15] data, where levels of adults’ physical activity during winter was less than the other seasons. A study in Iceland found that both older men and women were more active during summer than winter, with less time spent in accelerometer determined SB (4.4% in men, 2.5% in women) [14]. A study in the UK found that only time spent in LPA was significantly higher during spring than that during winter among older adults (winter: 2.7 ± 1.0 h/day, spring: 3.4 ± 1.3 h/day, summer: 3.1 ± 1.3 h/day, and autumn: 3.1 ± 1.2 h/day) [13]. We found that the magnitude of decline during winter seems to be large, compared to these Icelandic and UK studies. This may result from winter conditions, including amount of snowfall. Furthermore, our study participants were relatively more active and less sedentary during autumn, the non-snowfall season [35]. More highly active participants may experience activity decline to a greater degree, compared to low activity participants. In this sample, there were no significant seasonal changes of adherence to global physical activity guidelines. This may be due to very little time spent in MVPA in bouts of more than 10 min.

Gender differences of activity patterns were in line with our previous findings [35, 36] that Japanese older women were more physically active than older men, provided that all activity behaviors were assessed. In this sample, men experienced greater seasonal activity declines than did women. In Japan, women are more responsible for household chores and thus engage in a certain amount of activities throughout the year. There may be needed to develop a strategy to combat this seasonal decrease in physical activity for men.

In the current study, activity behavior patterns were unfavorably changed, with approximately 20% increase in SB and decrease in MVPA. The degree of the change in activity behaviors may be significant for health. A previous study found decline of physical activity during winter unfavorably affected the physical performance level after one year in community-dwelling older women, particularly its effect on maximum walking speed [37]. Given that the rate of muscle mass decline is higher in older adults compared to middle-aged adults [38], maintaining physical activity may be required to keep physical ability and prevent a negative cycle of frailty [39]. Another study showed short-term decreased physical activity with increased SB caused impairment of metabolism [40] which increases risk of cardiovascular disease [41]. Therefore, promoting physical activity during winter may be one way to tackle age-related diseases and loss of physical functioning.

### Strengths and limitations

Strengths of this study included objective assessment of activity behaviors and a novel statistical approach. Even though there has been increasing research using objective methods [13–15, 42], no study has treated with consideration to the co-dependence of time spent in activity behaviors. Moreover, we provided results from a seldom-studied elderly population in a rural snowfall area. However, several limitations should be considered to interpret the findings. We may under/overestimate of SB and LPA since Active style Pro cannot provide postural information. Also, some activities (e.g. snow removal and skiing) may be not captured accurately. Second, we may underestimate the decline of activity level since most of the days that participants wore an accelerometer were relatively good weather in spite of heavy snowfall area. It has previously been observed that longer day length is associated with increased device-based physical activity in the older population [42, 43]. Third, as Tokamachi city is a rural area where heavy snowfall is common during winter, it is not necessarily representative of Japanese rural areas with different climates. People in areas with less snowfall may experience a smaller decline of physical activity during snowfall season. More research is needed in the different climate zones from different geographic areas. Finally, selection bias may have occurred. In general, accelerometry respondents have been more active and healthier than non-participating older adults [44]. In this study, those had valid accelerometer data in snowfall season were more physically active than those who did not.

### Implications for future research, policy and practice

Our findings suggest several implications in terms of both development of interventions to protect against seasonal physical activity decline and physical activity surveillance/ monitoring. Further research regarding how to stay active during winter may be required for health promotion, particularly in regions that experience long winters and with severe weather (e.g. heavy snow). Given that approximately 60% of the land in Japan has snow and a cold winter and that a quarter of the population lives in those areas [45], leading an active lifestyle during winter potentially has a significant public health impact.

Since SB is the most affected by season, it may be better to focus on developing strategies to reduce time spent in sitting. Older adults might be particularly influenced by seasonal outside conditions due to reduced physical functioning and mobility, and spend much of their time indoors. It thus may be effective to provide supplementary resources for indoor activities (e.g. gymnastic exercise programs), sharing of household chores, and making educational/ instrumental supports for safe snow removal [46]. Further approach includes replacing mentally-passive with mentally-active SB may be effective for health, particularly for preventing cognitive decline [47, 48] and depression [49, 50].

As for physical activity surveillance/ monitoring, investigators should be aware of the potential for under- or overestimation of levels of activity especially when the interest is in its between-individual variation, including community-level and country-level comparisons. Seasonality also should be considered when intervention studies are performed.

## Conclusions

Accelerometer determined activity behaviors were greatly affected by season in community-dwelling older adults in a rural snowfall area, resulting in unfavorable changes, particularly in SB time, during snowfall season. Development of strategies to keep rural older adults active during the snowfall season may be needed for maintaining a consistently-active lifestyle for their health.

## Data Availability

The datasets used and analyzed during the current study are available from the corresponding author on reasonable request.

## Abbreviations

SB: Sedentary behavior
LPA: Light-intensity physical activity
MVPA: Moderate-to-vigorous physical activity
CoDa: Compositional data analysis
METs: Metabolic equivalents
